# N‐Geranylated Amino Acid Surfactants with Low Critical Micelle Concentrations from Abundant, Naturally Derived Starting Materials

**DOI:** 10.1002/open.202500421

**Published:** 2025-08-21

**Authors:** Brett L. Pollard, Yumeng Liu, Michael G. Gardiner, Luke A. Connal

**Affiliations:** ^1^ Research School of Chemistry The Australian National University Canberra ACT 2601 Australia

**Keywords:** aldehydes, amino acids, micelles, surfactants, sustainable chemistry

## Abstract

In this work, a simple method for the preparation of N‐alkylated amino acid surfactants in 1–2 steps is reported. These products perform comparably to existing ionic surfactants, with N‐tetrahydrogeranylated serine having a critical micelle concentration (CMC) of only 7.4 mmol·L^−1^. The suite of multiply charged surfactants generally possesses CMCs comparable to existing ionic surfactants. Further, their synthesis is simple, high‐yielding, scalable, and does not require complex purification. The most hydrophobic surfactant (N‐geranylated glycine) was found to have a log n‐octanol‐water partition coefficient (*P*_ow_) of 1.6 (indicating low bioaccumulative potential), and a single‐step product (N‐geranylated aspartic acid) was assessed for detergency potential via swatch testing.

## Graphical abstract

Cleaning Up Surfactants: Realising critical micelle concentrations lower than sodium dodecyl sulfate with sustainable surfactants prepared from amino acids and naturally occurring aldehydes.

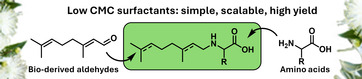

## 1 Introduction

Surface active agents, commonly contracted to “surfactants”, are near‐ubiquitous chemicals used both for domestic and industrial purposes including foaming, emulsifying, dispersing, and detergency. Now central to contemporary life, surfactants command a global market size of $66 billion USD at the time of publication.^[^ ^1^ ^]^ Hence, they become problematic when considering that ≈60% of the 15 million tons of surfactants used annually end up in the environment.^[^ ^2^ ^]^ This constitutes a substantial sink of largely petrochemically‐sourced carbon, as well as posing both human health concerns and serious ecological issues.^[^ ^3^ ^]^

Designing novel surfactants that are more environmentally benign, have more efficient syntheses, and use non‐petroleum derived starting materials has hence become a topic of great interest to the chemist. This is driven in no small part by commercial interest, with the international market for “green” surfactants being $2.5 billion USD (as of 2020) and growing with a compound annual growth rate of 5.7%.^[^ ^4^ ^]^ For this purpose, amino acids are a particularly attractive choice of hydrophilic head group,^[^ ^5^, ^6^ ^–^ ^7^ ^]^ being abundant, affordable, and biologically innocuous. This realization is not confined to academic research; in 2021, amino acid‐based surfactants represented over 20% of the green surfactant market share.^[^ ^4^ ^]^ These are typically modified to introduce a hydrophobic tail in one of two ways: either by functionalizing via esterification at the carboxylic acid, or through amination at the N‐terminus. This N‐terminus installation is traditionally performed using an alkyl halide and affords products with desirable surfactant properties.^[^ ^8^ ^–^ ^10^ ^]^

Rather than using petrochemically‐derived alkyl halides, we anticipated that biomass‐derived aldehydes, when reacted at the amino acid N‐terminus via a reductive amination, could provide a simpler route to otherwise inaccessible hydrophobic tails. This avoids the use of alkyl halides while retaining the benefits of a one‐pot preparation. In this work, we describe our efforts to prepare a suite of novel surfactants with gratifyingly low critical micelle concentrations (CMCs) using simple reductive amination protocols.

## 2 Results & Discussion

### 2.1 Synthesis

In selecting naturally occurring aldehydes, our key considerations were to select for naturally occurring platform molecules with relatively low toxicity, low cost, industrial scale availability, and those that were ideally biodegradable. A stand‐out option was 3,7‐dimethyl‐2,6‐octadienal (**1**), commonly referred to as citral or its two isomers, geranial and neral. Compound **1** is an α,β‐unsaturated aldehyde that also bears a dimethyl‐substituted alkene at the opposite end of the molecule. Its reduced form (and reductive amination side product) geraniol is also of low toxicity and is biodegradable, and so **1** was an obvious choice of hydrophobic tail for this work.

The first amino acid selected for optimizing the reaction was glycine, being the simplest amino acid and bearing no complicating R‐group nor stereocentre. However, glycine exhibits poor solubility in organic solvents under neutral to acidic conditions, massively limiting the efficiency of transformation. Indeed, our efforts to perform these reactions in acidic conditions offered dismal yields (typically < 30%). Hence, and contrary to conventional imine formation conditions, we opted to employ basic conditions previously leveraged by our group (using sodium hydroxide) to facilitate dissolution of the amino acid and so drive the reaction forward.^[^ ^11^ ^,^ ^12^ ^]^

**Scheme 1:**
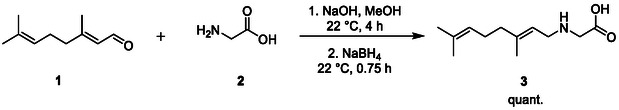
General reaction scheme for the synthesis of surface‐active N‐geranyl glycine **3** from citral (**1**) and glycine (**2**).

We found satisfactory formation of the imine in methanol at room temperature over a period of 4 h with the addition of a slight excess of NaOH (1.05 eq.). Reduction to the secondary amine via an excess of sodium borohydride (1.40 eq.) afforded, after a simple workup, the N‐geranylated glycine product **3** in quantitative yield and sufficient purity (**Scheme** **1**).

**Scheme 2:**
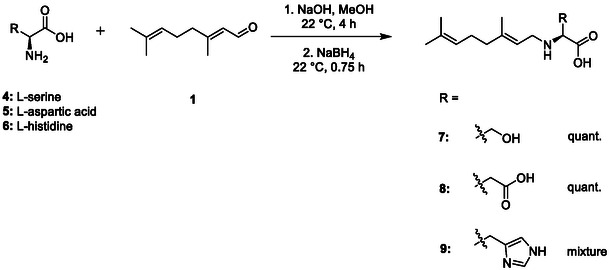
Efforts to expand the N‐alkylated amino acids library with different amino acids using reductive aminations in basic conditions.

We then extended these conditions to a range of other amino acids: L‐serine (**4**) (providing an additional hydrophilic ‐OH functionality), L‐aspartic acid (**5**) (providing an additional negative charge) and L‐histidine (**6**) (providing an additional positive charge) (**Scheme** **2**). N‐geranylated serine **7** and aspartic acid **8** products were obtained once again in quantitative yield and in sufficient purity, but selectivity issues were encountered in the formation of geranylated L‐histidine **9**. Analysis of the ^1^H‐NMR spectrum obtained for **9** indicated a side product formed by the nucleophilic addition of the imidazole nitrogen to the β‐position of citral. Performing this transformation instead in acidic conditions (in an effort to protonate the histidine nitrogen and hence reduce its nucleophilicity) still failed to afford the desired product **9** in sufficient purity.

**Scheme 3:**
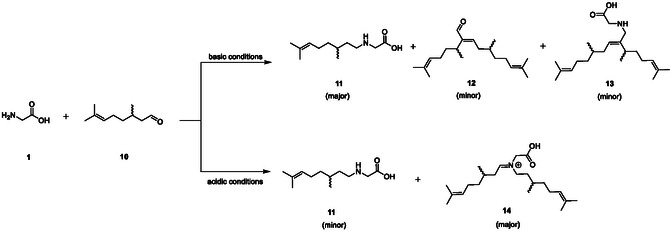
Undesirable side reactions of citronellal (**10**) in reductive aminations with glycine (**1**), where basic conditions afforded self‐aldol side products **12** and **13**, and acidic conditions primarily afford di‐addition to the amine (product **14**).

We next varied the hydrophobic tail, investigating citronellal (**10**), a similarly large scale, well‐studied and naturally occurring aldehyde, differing from citral (**1**) in that it lacks α,β‐unsaturation. Attempts to perform this reaction in the basic conditions successfully employed for **1** caused the formation of a mixture of aldol side products (**Scheme** **3**, top). We instead opted to employ more conventional acidic conditions using sulfuric acid. This hindered the dissolution of the various amino acids and hence yield (48%, as a mixture). We found that the product afforded was a mixture of iminium adduct **14** as the major product, as well as the desired product **11** (Scheme 3, bottom). Similar issues were observed in syntheses attempted using L‐serine (**4**) and L‐aspartic acid (**5**), which yielded analogous products and comparable yields.

Efforts were also made to prepare linear, saturated analogs using decanal. Again, basic reaction conditions were plagued with undesirable self‐aldol condensation side products which necessitated complex purifications that were not pursued. Acidic conditions afforded the desired products, although these were heavily contaminated with decanol as a major side product. Employing an excess of the amino acids improved product yield, but any practical efforts to separate residual decanol from the desired compounds proved unsatisfactory. While these products would likely make excellent surfactants, their difficulty in purification made them undesirable targets.

**Scheme 4:**
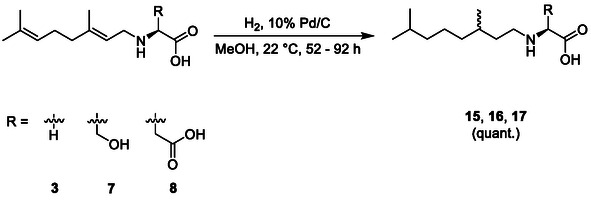
Hydrogenation of N‐alkylated amino acids **3**, **7**, and **8** employing hydrogen gas with palladium on carbon and affording products **15**, **16**, and **17** in quantitative yield.

In an effort to extend the series with industrially relevant transformations, we next looked to reduce the geranylated species using hydrogen and palladium on carbon. These transformations proved effective and easy, affording compounds **15**, **16**, and **17** once more in quantitative yield after simple purifications (**Scheme** **4**).

### 2.2 Determination of Critical Micelle Concentrations

**Figure 1:**
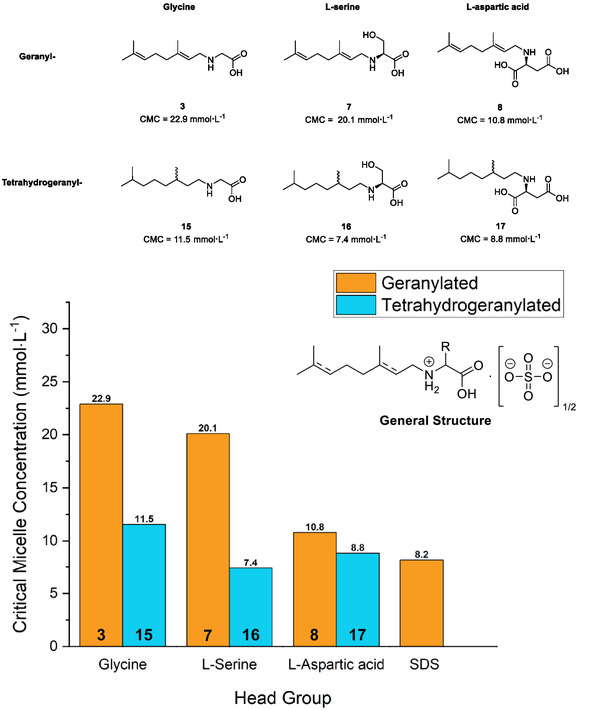
Top: Suite of analogs prepared with CMC values also provided. Bottom: CMC values obtained for various products expressed in a bar graph, showing both the effect of modifying the head group and tail group, as well as the degree of saturation on the tail installed (compound numbers are shown within their respective bar). General structure with counter ion is shown top right.

In order to study the surface activity of the products obtained, we investigated the CMCs of the products involved using pendant drop tensiometry. Briefly, this method involves measuring the interfacial contact angle between a drop of analyte solution (typically aqueous) and another medium (typically air), where saturation of the interface by surfactant molecules renders micellization thermodynamically favorable. A plot of recorded surface tension versus the binary logarithm of analyte concentration (here calculated as a molecule of surfactant with one half of a sulfate anion) will afford two linear regions, the intersection of which is the CMC. The results of these measurements are given below in **Figure** **1**.

As we anticipated, the zwitterionic surfactant **3** showed the highest CMC value of the series, with a CMC of 22.9 mmol·L^−1^. Geranylated L‐serine (**7**) performed marginally better (CMC = 20.1 mmol·L^−1^) owing to the increased hydrophilicity of the headgroup. The addition of an additional carboxylic acid in product **8** gave a net negatively charged surfactant and markedly improved surface activity, with a CMC of 10.8 mmol· L^−1^. Complete reduction of the geranyl tail to tetrahydrogeranyl resulted in improved surface activity for all analogs. This was most apparent for compounds **15** and **16** which now exhibited CMC values of 11.5 and 7.4 mmol·L^−1^ respectively (50 and 63% lower than the geranyl analogs). This observation matched our expectations, with the removal of the rigid alkene units enhancing free rotation and hence stacking of the hydrophobic tails. These values were also gratifyingly similar to that of the traditional anionic surfactant sodium dodecyl sulfate, which exhibits a typical CMC value of ≈8.2 mmol·L^−1^. Although our synthetic approach failed to afford the citronellal‐substituted amino acids in appropriate yield, based on these results we anticipate that these would have exhibited CMC values lying between those of the geranylated and tetrahydrogeranylated analogs given their mono‐unsaturated tails.

### 2.3 Octanol‐Water Partition Coefficient

The n‐octanol‐water partition coefficient (*P*_ow_) is the commonly accepted measure of an analyte's lipophilicity and consequent bioaccumulation potential in animal fatty tissue. Analytes with a log *P*_ow_ value greater than 5 are generally considered to bioaccumulate and may pose a threat as persistent organic pollutants.^[^ ^13^ ^]^ In order to assess the bioaccumulative potential of our reported surfactants, we conducted a *P*_ow_ determination using the shake flask method.^[^ ^14^ ^]^ While the shake flask method is not typically appropriate for surfactant analytes, it has been successfully employed by other researchers^[^ ^9^ ^]^ and we found it to be the most rigorous technique possible with our available equipment. We selected our most lipophilic analyte (geranylated glycine **3**) to represent the “worst case” sample and performed analyses using ^1^H‐NMR spectroscopy in deuterium oxide with *N,N‐*dimethylformamide (DMF) as an internal reference. Taking the average concentration values for a series of differing n‐octanol‐water volume ratios gave a mean log *P*_ow_ of 1.6, which was far below the threshold value of 5.

### 2.4 Detergency Swatch Test

**Figure 2:**
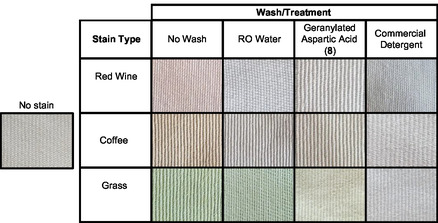
Swatch test performed with cotton‐polyester swatches stained with red wine, coffee or grass, washed with either RO water, commercial detergent (Cold Power Advanced Clean) or geranylated aspartic acid (**8**).

A swatch test was also conducted to assess the detergent activity of our most industrially relevant surfactant, geranylated aspartic acid (**8**). Compound **8** exhibited the lowest CMC value of the single‐step preparation surfactants and hence made an ideal candidate for a swatch test. Swatches cut from a clean, prewashed laboratory coat (cotton‐polyester blend) were stained with either red wine, coffee or grass, allowing the stains to set and dry for 24 h. These were then washed with either RO water, compound **8** (3.86 g·L^−1^ in RO water), or commercial detergent (Cold Power Advanced Clean) at the manufacturer's recommended concentration of 5 g·L^−1^ in Falcon tubes at 22 °C for 1 h with mechanical agitation. Swatches were then briefly rinsed in RO water and allowed to thoroughly air dry before imaging, with the results presented below in **Figure** **2**.

Across the stains tested, the commercial (enzymatic) detergent performed excellently, as would be expected for a specially formulated cold washing laundry detergent. All wash conditions, including RO water, performed remarkably well against the red wine stain. Against coffee, we observed that the commercial detergent showed excellent detergency, while RO water and compound **8** performed similarly. The grass stain was minimally affected by washing with RO water, but washing with compound **8** resulted in a marked decrease in stain color intensity. The commercial detergent appeared to completely remove the grass stain.

## 3 Conclusions

A suite of surface‐active compounds has been synthesized using bio‐derived platform molecules in high or quantitative yields using simple, industrially relevant transformations and purifications. In particular, products **8** and **15—17** were found to exhibit excellent surface activity as assessed using surface tensiometry. The reductive amination protocol was simple and efficient for preparing analogous structures and enabled access to industrially significant, naturally occurring aldehydes. While certain aldehydes afforded multi‐tailed mixtures, we anticipate that these still exhibit potentially useful surfactant properties. This method would enable access to an extremely wide range of compounds, and future studies in both the toxicity and environmental fate of these surfactants would elucidate their potential for wider applications.

## 4 Experimental Section

4.1

4.1.1

##### Chemicals and Reagents

Starting materials and reagents were generally available from Sigma–Aldrich, Merck, TCI, Strem or Lancaster Chemical Companies and were used as supplied. Drying agents and other inorganic salts were purchased from the AJAX, BDH, Chemsupply, or Unilab Chemical Companies. Where necessary, reactions were performed under an inert atmosphere.

##### Characterization

Unless otherwise specified, proton (^1^H) and carbon (^13^C) NMR spectra were recorded at 22 °C in (CD_3_)_2_SO on a Varian spectrometer operating at 400 MHz for proton and 100 MHz for carbon nuclei. The signal due to residual DMSO appearing at *δ*H 2.50 and the central resonance of the DMSO “triplet” appearing at *δ*C 39.5 were used to reference ^1^H and ^13^C NMR spectra, respectively. All NMR spectra are provided in the Figure S2–S14, Supporting Information. Infrared spectra (*ν*_max_) were recorded on a Perkin‐Elmer 1800 Series FTIR Spectrometer. Samples were analyzed as thin films on KBr plates. Low‐resolution electrospray ionization (ESI) analyses were carried out on an Agilent 6120 Quadrupole LC‐MS instrument connected to an Agilent 1200 series LC unit. An isocratic elution mode was used to deliver the sample into the mass spectrometer. Capillary voltage was set at 5000 V and the desolvation temperature was set at 300 °C, using atmospheric pressure ionization. MSDChem software was used for data analyses. HR ESI analyses were performed on a Waters Synapt G2‐Si HDMS qTOF (time of flight) mass spectrometer connected to a Waters Acquity UPLC I Class plus LC unit. An isocratic elution mode was used to deliver the sample (without chromatographic separation) using methanol as the eluent at a flow rate of 0.2 mL min^−1^. The mass spectrometer was operated in positive fullMS resolution and TOF modes. Capillary and cone voltages were set at 2 and 30 V, respectively. The source temperature was set at 120 °C and the desolvation temperature was set at 200 °C. Leucine enkephalin was used as the lockspray reference compound. Elemental composition reports were produced within 3‐ppm error. MassLynx software (ver. 4.1) was used to process data. HR ESI experiments were also performed using an Orbitrap Elite mass spectrometer equipped with a HESI‐II ESI source coupled to an Ulti‐Mate 3000 UHPLC (Thermo Scientific). Samples were introduced by direct infusion without chromatographic separation. Scans were performed on the orbitrap FTMS mass analyzer at a resolution of 120 000 (m/z 200–2000). The spray voltage was set at 4.3 kV and the source heater temperature at 300 °C. The data were analyzed using Thermo Freestyle software (see https://www.thermofisher.com/hk/en/home/technical‐resources/technical‐reference‐library/mass‐spectro‐metry‐support‐center/liquid‐chromatography‐mass‐spectro‐metry‐software‐support/freestyle‐software‐support.html). Melting points are uncorrected. Optical rotations were recorded in the indicated solvent at 20 °C on a Perkin‐Elmer Model 343 Polarimeter. Suitable crystals were selected and the crystals mounted on MiTeGen holders in oil on a SuperNova, Dual, Cu at home/near, HyPix diffractometer. The crystals were kept at 150.01(10) K during data collection. Data was collected and processed with CrysAlisPro Software System, Rigaku Oxford Diffraction (2022). Using Olex2,^[^ ^15^ ^]^ the structures were solved with the SHELXT^[^ ^16^ ^]^ structure solution program using Intrinsic Phasing and refined with the SHELXL^[^ ^17^ ^]^ refinement package using Least Squares minimisation. The hydrocarbyl chain ends were found to be highly disordered, with one of the positional occupancies of each included in the refinement model explicitly. Refer to the _refine_special_details field of the CIF file for detailed handling of the refinement. Structure of the sulfate salt of 3 is given in the Figure S1, Supporting Information.

The surface tensions of surfactant solutions were measured by the pendant drop method using a CAM200 KSV contact angle goniometer at a temperature of 25 ± 1.0 °C and with all analyte solutions maintained at pH 7. Measurements were repeated a minimum of five times, and the average value was reported. The estimated error in the reported values is ±0.1 mNm^−1^. Plotting the average measurements obtained against the log_2_ of the concentration and finding the intercept of the two lines thus obtained was used to determine CMCs. Plotted data is provided in the Figure S15–S21, Supporting Information.

The *P*_ow_ was determined using the shake flask method, with all protocols being performed at 22 °C (±1 °C).^[^ ^14^ ^]^ Equal volumes of deuterium oxide and n‐octanol were intimately mixed and left to equilibrate for 48 h. A stock solution of the analyte was then prepared using this n‐octanol saturated D_2_O and a reference ^1^H‐NMR spectrum recorded. Aliquots (500 µL) of this analyte stock were then transferred into screwcap vials before being treated with n‐octanol in solvent ratios of 1:2, 1:1, and 2:1 for n‐octanol:D_2_O, respectively. These mixtures were then intimately mixed and left to equilibrate for 72 h. Equivalent volumes of the aqueous phases were then collected and treated with a stock solution of DMF in D_2_O, mixed, and analyzed by ^1^H‐NMR spectroscopy. Relative integrations of the reference (DMF) to analyte were then used to determine the concentration of analyte remaining dissolved in the aqueous phase. *P*_ow_ values were then calculated as follows:

(1) $$P_{\mathrm{ow}}=\log_{10}\;\left(\frac{c_{n-\text{octanol}}}{c_{\text{water}}}\right)$$

Swatch testing was performed using cotton‐polyester swatches taken from a prewashed and dried laboratory coat and were cut into equal sections size, 30 × 60 mm. Test swatches were then soaked in one of the following: red wine (pinot noir), coffee (espresso), or grass (extract from mechanically ground foliage) and left to air dry at 22 °C for 24 h. Then, in separate Falcon conical centrifuge tubes, the swatches were submerged in 40 mL of either: reverse osmosis (RO) water, analyte **8** in RO water (3.86 g·L^−1^) or commercial detergent at the manufacturer's recommended concentration for hand washing (Cold Power Advanced Clean, 5 g·L^−1^ in RO water) before agitation at 22 °C for 1 h. Swatches were then removed, briefly rinsed in fresh RO water and left to air dry for 24 h before imaging. Swatches were photographed using identical lighting and magnification and without any adjustments or image manipulation. The resulting images were used to visually compare test swatches to the relevant controls.

##### Synthesis of (3,7‐Dimethylocta‐2,6‐Dien‐1‐Yl)glycine (3)

To a magnetically stirred solution of glycine (1.00 g, 13.3 mmol) and sodium hydroxide (568 mg, 14.2 mmol) in methanol (157 mL) at 22 °C was added E/Z citral (2.23 g, 2.50 mL, 14.7 mmol). After 4 h, sodium borohydride (808 mg, 21.3 mmol) was added in portions to the clear, light‐yellow solution. The ensuing mixture was stirred for an additional 0.5 h before quenching with conc. sulfuric acid until pH ≈ 7. The resulting suspension was then gravity filtered, and the filtrate obtained concentrated under reduced pressure to afford a yellow oil (quant.) which was used for surface tensiometry studies. Further washing of the oil with diethyl ether (5 × 20 mL) afforded **3** as an off‐white powder (49%); m.p. 218 °C (decomp.); ^1^H NMR (400 MHz, CD_3_OD) *δ* 5.26 (t, *J* = 7.0 Hz, 1H), 5.15—5.08 (m, 1H), 3.28—3.21 (m, 2H), 3.19—3.13 (m, 2H), 2.15—2.01 (m, 4H), 1.74 (s, 1H), 1.67 (s, 5H), 1.61 (s, 3H); ^13^C NMR (101 MHz, CD_3_OD) *δ* 177.8, 140.9, 132.5, 125.1, 121.9, 52.6, 47.1, 40.8, 27.5, 23.7, 17.7, 16.4; IR (KBr) *ν*_max_ 3385, 2967, 2914, 2855, 1714, 1627, 1436, 1376, 1220, 1059, 1014, 920, 832, 753, 611 cm^−1^; HRMS (ESI, –ve) m/z (M–H)^‐^ calculated for C_12_H_20_NO_2_ 210.1500; found 210.1506.

##### Synthesis of (3,7‐Dimethylocta‐2,6‐Dien‐1‐Yl)‐L‐Serine (7)

To a magnetically stirred solution of L‐serine (1.00 g, 9.55 mmol) and sodium hydroxide (400 mg, 10.0 mmol) in methanol (112 mL) at 22 °C was added E/Z citral (1.59 g, 1.78 mL, 10.5 mmol). After 4 h, sodium borohydride (578 mg, 15.3 mmol) was added in portions to the clear, light‐yellow solution. The ensuing mixture was stirred for an additional 0.5 h before quenching with conc. sulfuric acid until pH ≈ 7. The resulting suspension was then gravity filtered, and the filtrate obtained concentrated under reduced pressure to afford compound **33** as a white powder (quant.); m.p. 149–151 °C; [*α*]_D_ –4.00 (*c* = 0.2, CH3OH). ^1^H NMR (400 MHz, CD_3_OD) *δ* 5.31 (t, *J* = 7.5 Hz, 1H), 5.15—5.08 (m, 1H), 4.00—3.94 (m, 1H), 3.89—3.82 (m, 1H), 3.76–3.68 (m, 2H), 3.57–3.51 (m, 1H), 2.20–2.08 (m, 4H), 1.83 (s, 1H), 1.75 (s, 2H), 1.68 (s, 3H), 1.62 (s, 3H); ^13^C NMR (101 MHz, CD_3_OD) *δ* 171.4, 147.3, 133.0, 124.7, 115.4, 63.8, 61.2, 45.0, 40.7, 33.1, 27.2, 25.9, 17.7, and 16.7; IR (KBr) *ν*_max_ 3287, 2965, 2914, 2855, 1641, 1591, 1437, 1404, 1375, 1223, 1065, 1008, 769, 680, and 572 cm^−1^; HRMS (ESI, –ve) m/z (M–H)^‐^ calculated for C_13_H_22_NO_3_ 240.1605; found 240.1607.28

##### Synthesis of (3,7‐Dimethylocta‐2,6‐Dien‐1‐Yl)‐L‐Aspartic Acid (8)

To a magnetically stirred solution of L‐aspartic acid (1.00 g, 7.52 mmol) and sodium hydroxide (605 mg, 15.1 mmol) in methanol (88 mL) at 22 °C was added E/Z citral (1.26 g, 1.41 mL, 8.26 mmol). After 4 h, sodium borohydride (457 mg, 12.1 mmol) was added in portions to the clear, light‐yellow solution. The ensuing mixture was stirred for an additional 0.5 h before quenching with conc. sulfuric acid until pH ≈ 7. The resulting suspension was then gravity filtered, and the filtrate obtained concentrated under reduced pressure to afford compound **34** as a waxy yellow solid (quant.); [*α*]_D_ −8.98 (*c* = 0.2, CH_3_OH). ^1^H NMR (400 MHz, CD_3_OD) *δ* 5.38—5.29 (m, 1H), 5.15—5.07 (m, 1H), 3.74 (s, 2H), 3.69 (s, 1H), 3.07—2.76 (m, 2H), 2.21—2.07 (m, 4H), 1.82 (d, *J* = 5.0 Hz, 1H), 1.77 (s, 2H), 1.68 (s, 3H), 1.62 (s, 3H); ^13^C NMR (101 MHz, CD_3_OD) *δ* 177.6, 172.6, 147.4, 133.0, 124.6, 115.2, 52.7, 45.9, 40.7, 35.4, 27.2, 25.8, 23.8, and 17.8; IR (KBr) *ν*_max_ 3385, 2967, 2916, 2856, 1736, 1626, 1438, 1376, 1208, 1059, 1011, 833, 759, 643, and 531 cm^−1^; HRMS (ESI, –ve) m/z (M–H)^−^ calculated for C_14_H_22_NO_4_ 268.1554; found 268.1556.

##### Synthesis of (3,7‐Dimethyloctyl)glycine (15)

A magnetically stirred solution of compound **3** (1.40 g, 6.63 mmol) in methanol (50 mL) was treated with 10% palladium on carbon (138 mg) and placed under a balloon of hydrogen. After 52 h, the reaction mixture was filtered through a pad of diatomaceous earth, and the filtrate concentrated under reduced pressure to afford **15** as a waxy yellow solid (quant.); [*α*]_D_ +0.71 (*c* = 0.1, CH_3_OH). ^1^H NMR (400 MHz, CD_3_OD) *δ* 3.18 (s, 2H), 2.71—2.58 (m, 2H), 1.62—1.44 (m, 3H), 1.40–1.25 (m, 4H), 1.22–1.12 (m, 3H), 0.89 (t, *J* = 8.4 Hz, 9H); ^13^C NMR (101 MHz, CD_3_OD) *δ* 177.3, 53.4, 48.3, 40.5, 38.5, 37.1, 32.2, 29.1, 25.8, 23.1, 23.0, and 20.0; IR (KBr) *ν*_max_ 3396, 2955, 2923, 2869, 1600, 1531, 1476, 1413, 1384, 1328, 1285, 928, 699, 652, and 560 cm^−1^; HRMS (ESI, –ve) m/z (M–H)^‐^ calculated for C_12_H_24_NO_2_ 214.1807; found 214.1814.

##### Synthesis of (3,7‐Dimethyloctyl)‐L‐Serine (16)

A magnetically stirred solution of compound **7** (958 mg, 3.97 mmol) in methanol (50 mL) was treated with 10% palladium on carbon 140 mg) and placed under a balloon of hydrogen. After 68 h, the reaction mixture was filtered through a pad of diatomaceous earth, and the filtrate concentrated under reduced pressure to afford **16** as a white powder (quant.); m.p. 140–142 °C; [*α*]_D_ –4.79 (*c* = 0.2, CH_3_OH). 1H NMR (400 MHz, CD_3_OD) *δ* 4.03—3.83 (m, 2H), 3.69 (s, 1H), 3.55 (dd, *J* = 6.5, 3.8 Hz, 1H), 3.22 (d, *J* = 2.7 Hz, 1H), 3.18—2.97 (m, 2H), 1.82—1.69 (m, 1H), 1.62—1.48 (m, 3H), 1.41—1.26 (m, 3H), 1.25—1.13 (m, 3H), 0.92 (dd, *J* = 21.1, 6.5 Hz, 9H); ^13^C NMR (101 MHz, CD_3_OD) *δ* 65.4, 61.2, 46.4, 40.4, 38.2, 34.4, 32.1, 29.1, 25.7, 23.0, and19.6 (low s/n, carboxylic acid signal 29 not found); IR (KBr) *ν*_max_ 3264, 2927, 2867, 1639, 1592, 1557, 1497, 1459, 1433, 1385, 1366, 1332, 1219, 1162, and1065 cm^−1^; HRMS (ESI, –ve) m/z (M–H)^‐^ calculated for C_13_H_26_NO_3_ 244.1913; found 244.1925.

##### Synthesis of (3,7‐Dimethyloctyl)‐L‐Aspartic Acid (17)

A magnetically stirred solution of compound **8** (595 mg, 2.21 mmol) in methanol (20 mL) was treated with 10% palladium on carbon (78 mg) and placed under a balloon of hydrogen. After 92 h, the reaction mixture was filtered through a pad of diatomaceous earth, and the filtrate concentrated under reduced pressure to afford **17** as a waxy yellow solid (quant.); [*α*]_D_ −14.54 (*c* = 0.2, CH3OH). ^1^H NMR (400 MHz, CD_3_OD) *δ* 3.74 (s, 1H), 3.69 (s, 1H), 3.25—2.68 (m, 4H), 1.83—1.69 (m, 1H), 1.55 (p, *J* = 6.8 Hz, 3H), 1.40—1.26 (m, 3H), 1.24—1.13 (m, 3H), 0.92 (dd, *J* = 23.0, 5.6 Hz, 9H); ^13^C NMR (101 MHz, CD_3_OD) *δ* 173.3, 172.8, 55.1, 47.2, 46.0, 40.3, 38.0, 35.6, 34.7, 34.4, 31.9, 29.1, 25.7, 23.1, 23.0, and19.6; IR (KBr) *ν*_max_ 3462, 2953, 2927, 2869, 1745, 1586, 1384, 1300, 1216, 1170, 1064, 1013, 849, 767, and558 cm^−1^; HRMS (ESI, –ve) m/z (M–H)^−^ calculated for C_14_H_26_NO_4_ 272.1862; found 272.1871.

## Conflict of Interest

The authors declare no conflict of interest.

## Supporting information

Supplementary Material

## Data Availability

The data that support the findings of this study are available in the supplementary material of this article.
