# Supplementary material for: N‐Geranylated Amino Acid Surfactants with Low Critical Micelle Concentrations from Abundant, Naturally Derived Starting Materials
Source: ChemistryOpen. 2025 Aug 21;14(12):e202500421. doi: 10.1002/open.202500421 (PMC12680550; doi:10.1002/open.202500421)
Supplement: Supplementary file 1 — Supplementary Material [file OPEN-14-e202500421-s001.pdf]

N-geranylated amino acid surfactants with low CMCs from abundant, naturally derived starting materials

Brett L. Pollard,*,† Yumeng Liu,† Michael G. Gardiner, Luke A. Connal*

[a] Research School of Chemistry, The Australian National University, Canberra, ACT 2601, Australia.

†Authors contributed equally to this work.

* Corresponding Author

Supporting Information

Table of Contents

Plot derived from the single-crystal X-ray analyses of sulfate salt of compound **3**……..…...2

^{1}H and ^{13}C{^{1}H}NMR spectra……………………………………………………..…...3

Surface tensiometry data…………………………………………………………………...16

Single crystals of the sulfate salt of **3** were grown from acetone as colourless plate-shaped crystals $C_{24}H_{46}N_2O_8S$, (M = 522.69 g/mol): monoclinic, space group $P2_1/c$ (No. 14), a = 19.1605(4) Å, b = 18.9086(3) Å, c = 16.0063(2) Å, β = 90.8328(14)°, $\alpha = \gamma$ = 90°, V = 5798.45(16) Å^3, Z = 8, Z' = 2, T = 150.00(10) K, μ(Cu Kα) = 1.298 mm^{-1}, D_{calc} = 1.006 g/cm^3, 37449 reflections measured (3.284° ≤ 2θ ≤ 67.724°), 10463 unique (R_{int} = 0.0517, R_{sigma} = 0.0493) which were used in all calculations. The final R_1 was 0.0910 (I ≥ 2σ(I)) and wR_2 was 0.3030 (all data).

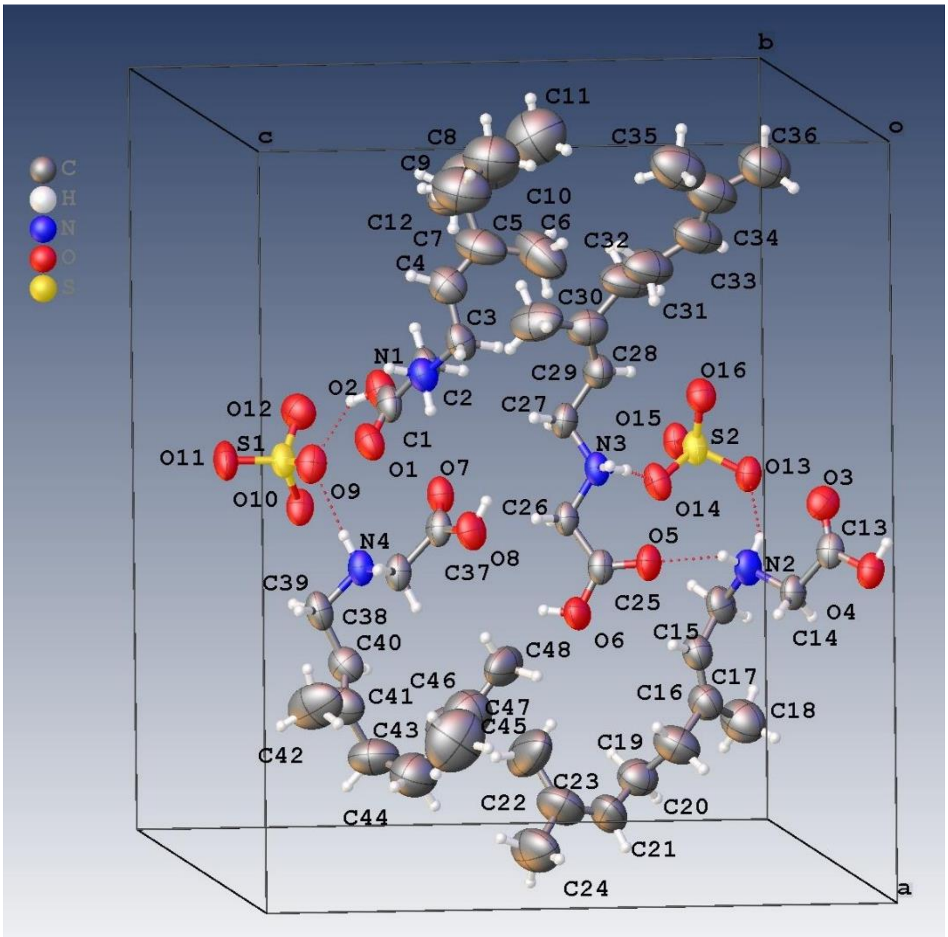

Figure S1: Structure of the sulfate salt of **3** showing the asymmetric unit (atomic displacement parameters shown at 50% probability level, each of the organic cations displayed poorly ordered chain ends and the figure shows only the 31-60% occupancy positions of the disorder component of the terminal 5 or 6 C atom chain ends that were included in the refinement model, with a solvent mask used to handle other void space in this region).

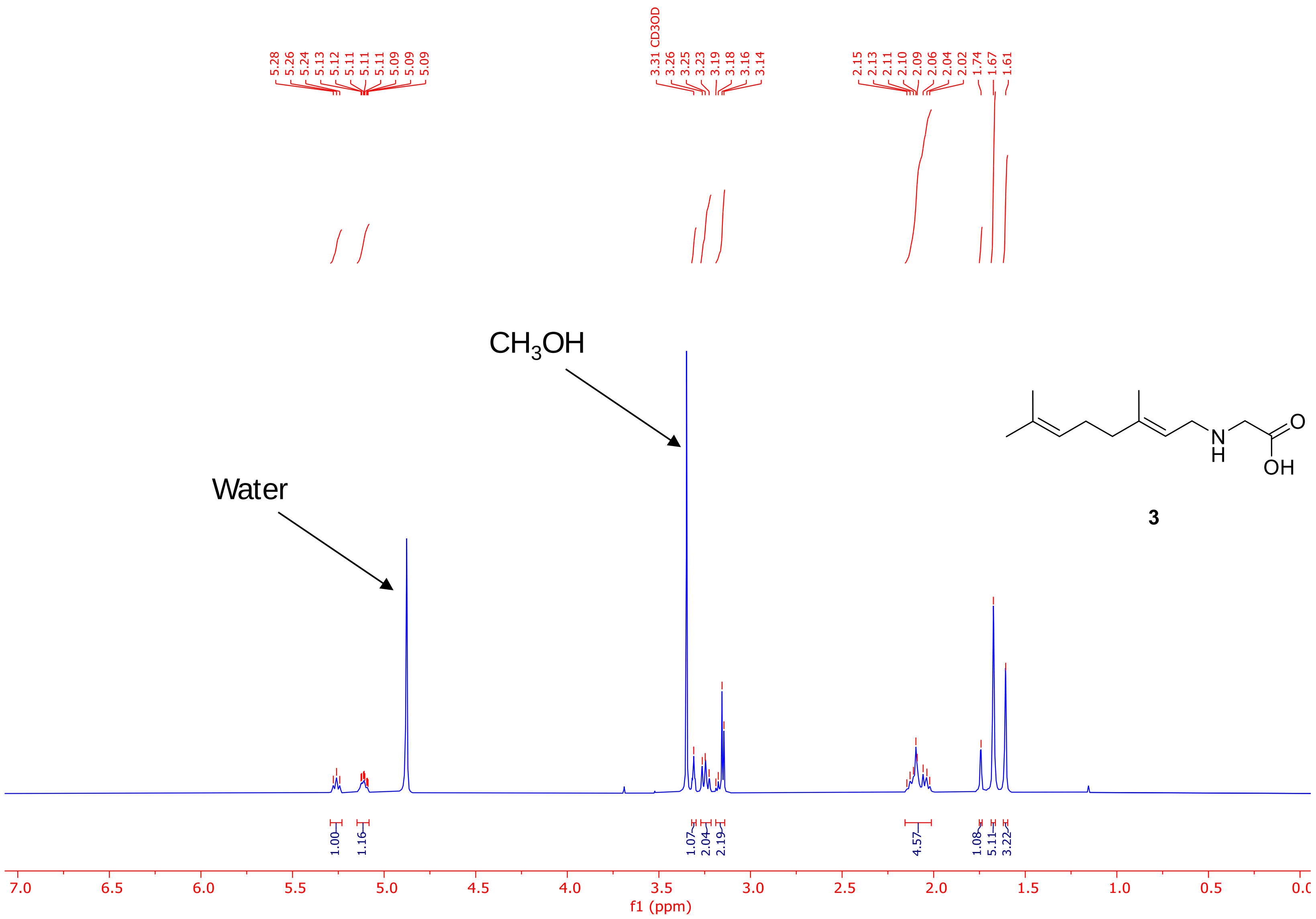

Figure S2: 400 MHz ^{1}H NMR spectrum [recorded in (CD_3OD)] of compound **3**.

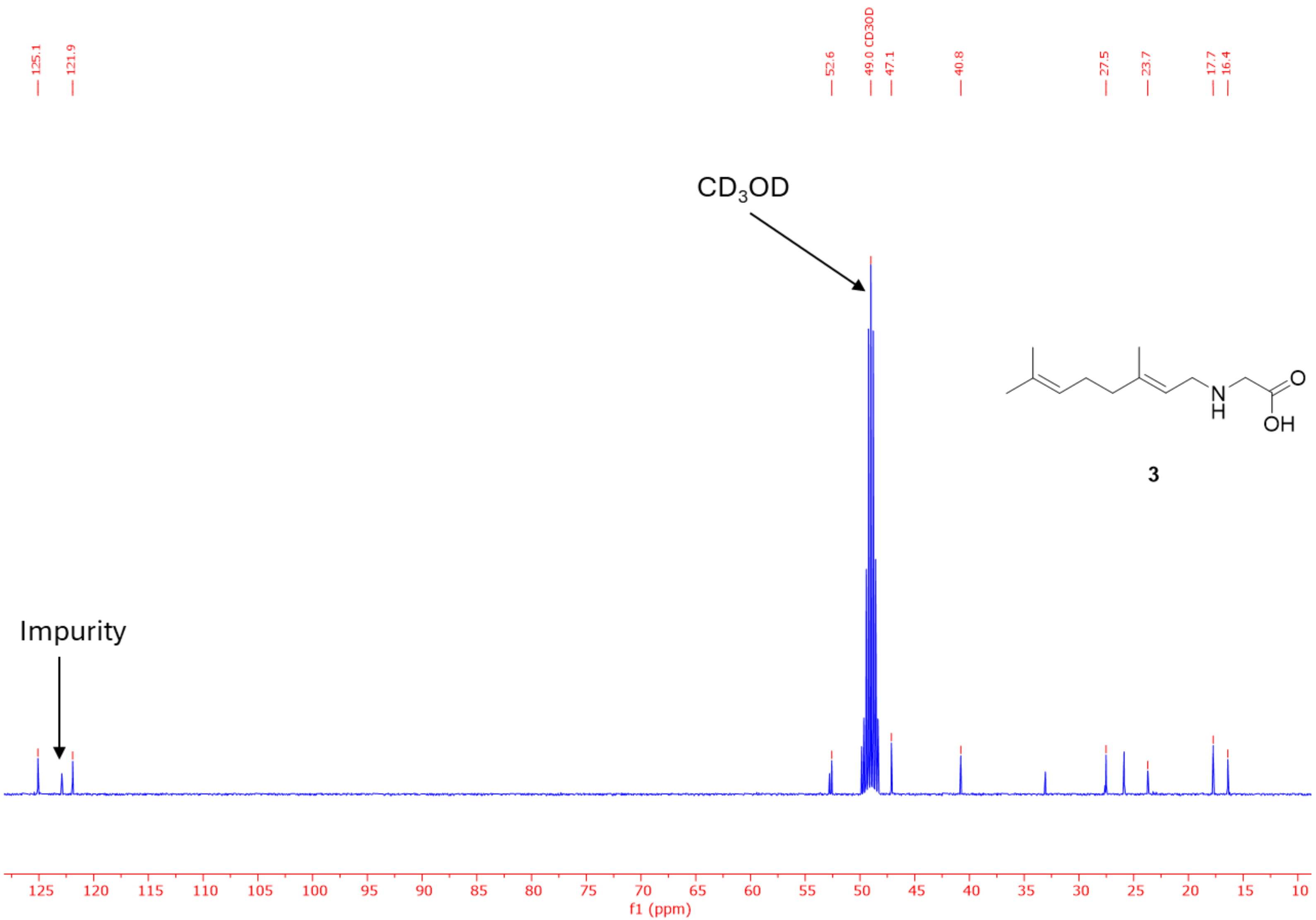

Figure S3: 101 MHz $^{13}C\{^{1}H\}$ NMR spectrum [recorded in (CD_3OD)] of compound **3**. Additional peaks arising due to isomerism and impurities carried through from the starting material.

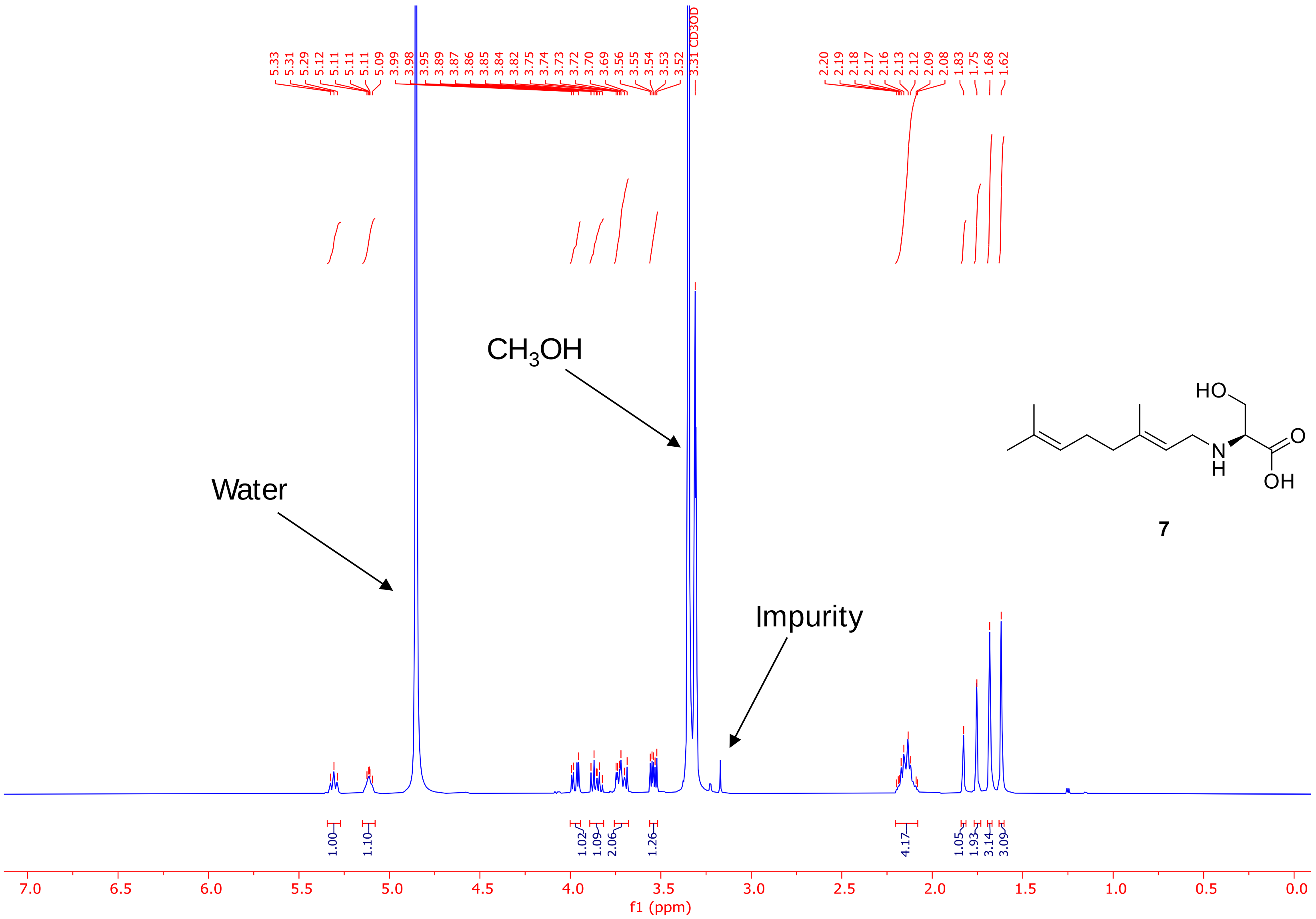

Figure S4: 400 MHz ^{1}H NMR spectrum [recorded in (CD_3OD)] of compound **7**.

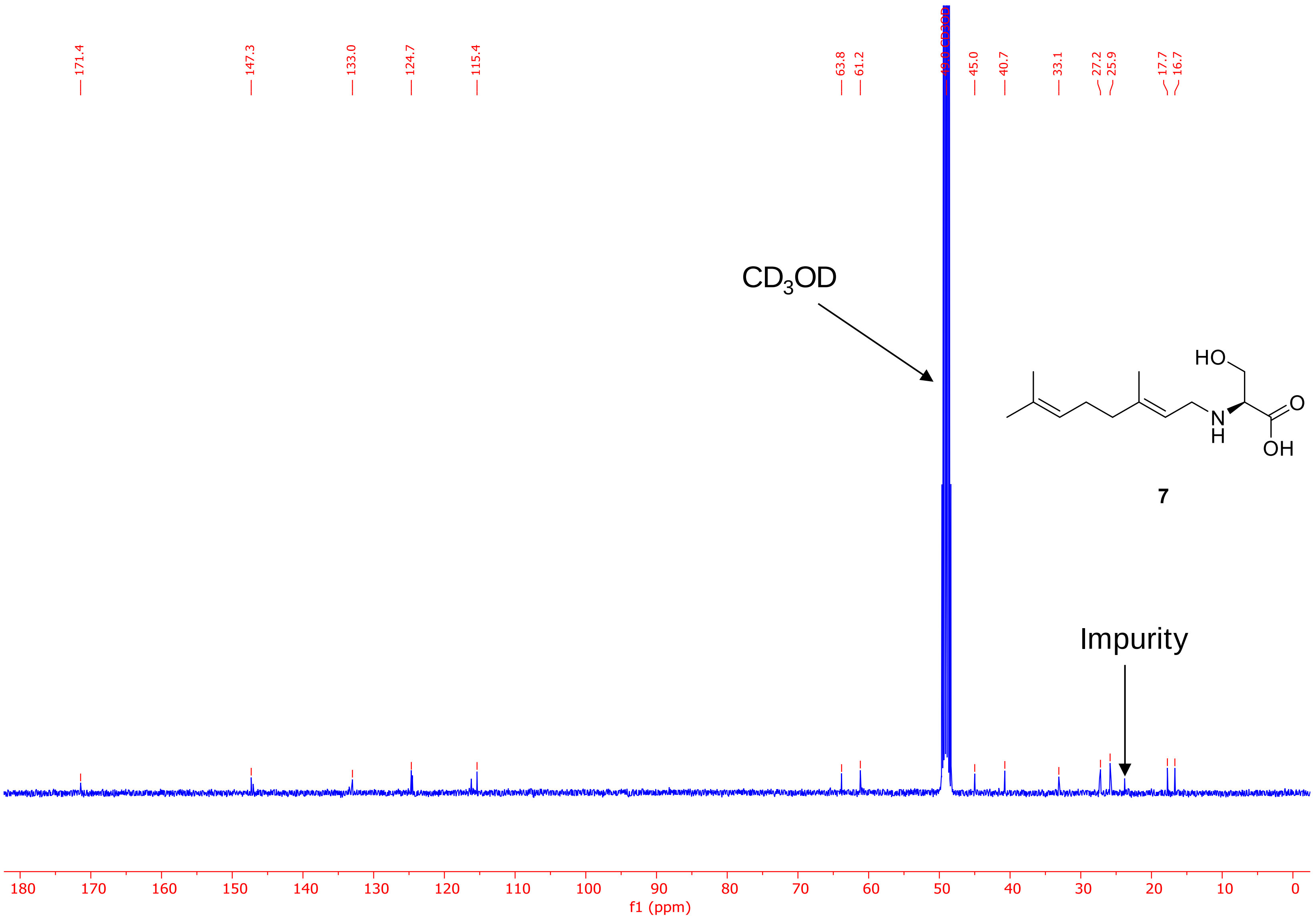

Figure S5: 101 MHz $^{13}C\{^1H\}$ NMR spectrum [recorded in (CD_3OD)] of compound **7**.

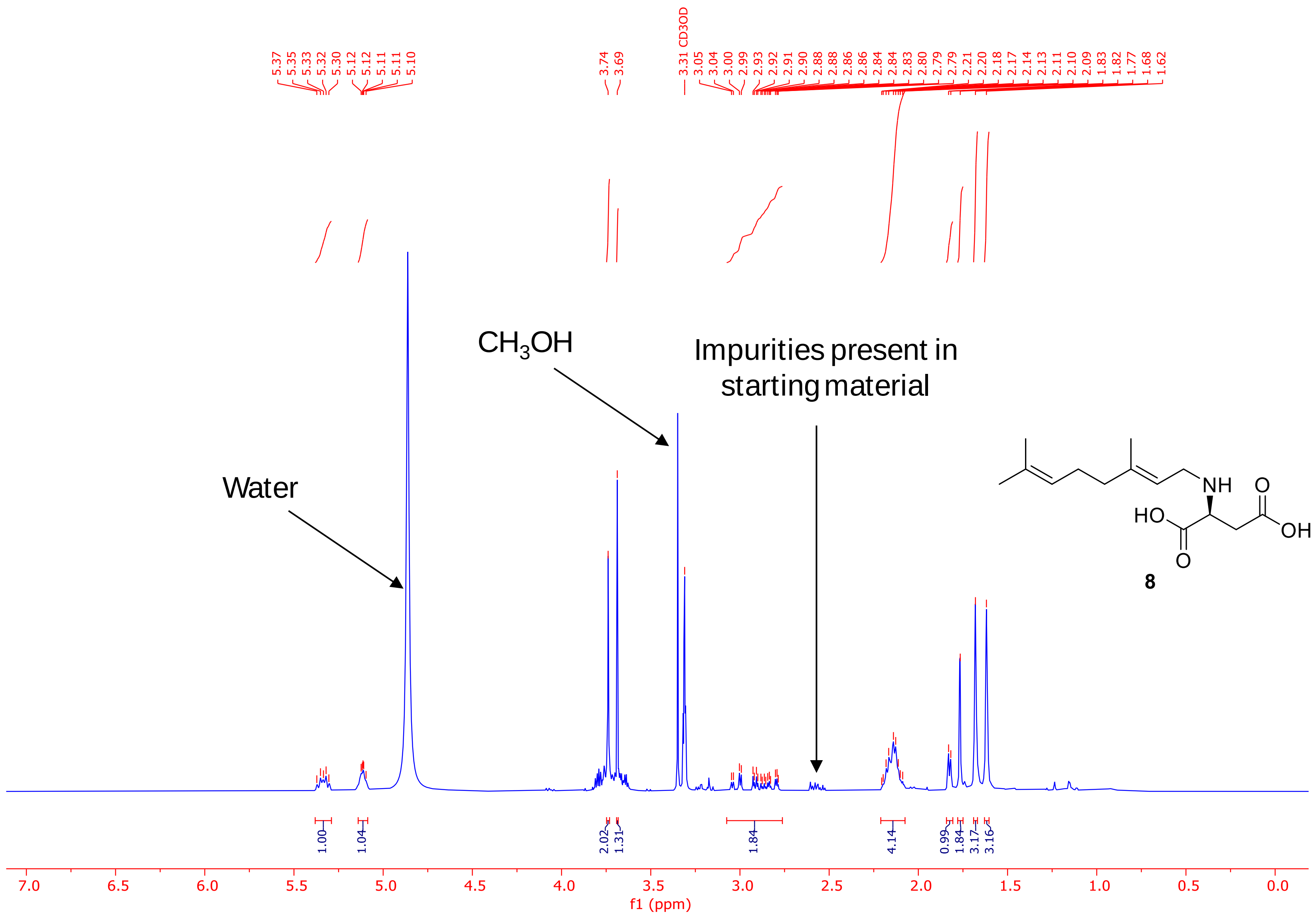

Figure S6: 400 MHz ^{1}H NMR spectrum [recorded in (CD_3OD)] of compound **8**.

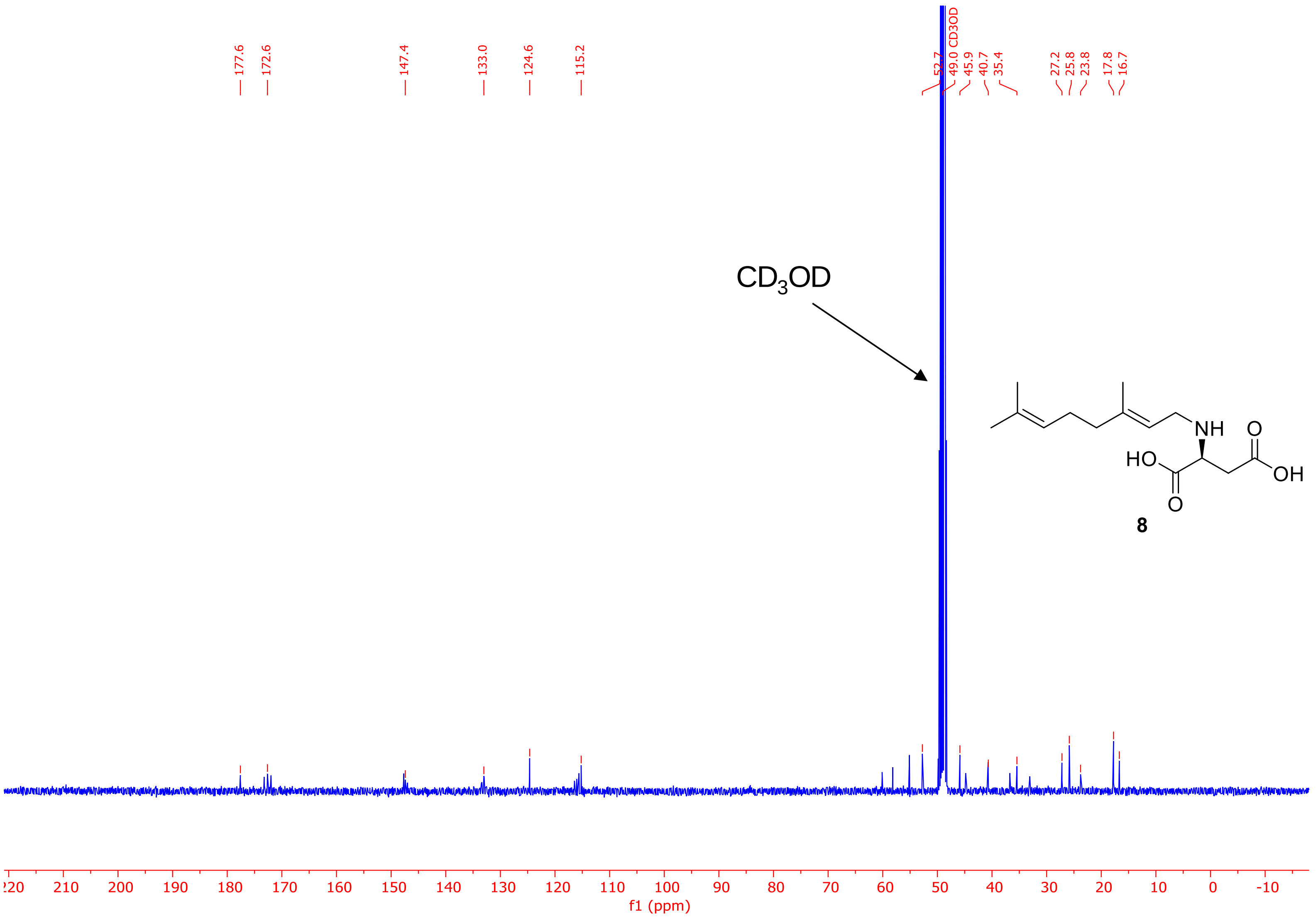

Figure S7: 101 MHz $^{13}C\{^{1}H\}$ NMR spectrum [recorded in (CD_3OD)] of compound **8**. Additional peaks arising due to isomerism and impurities carried through from the starting material (*L*-aspartic acid).

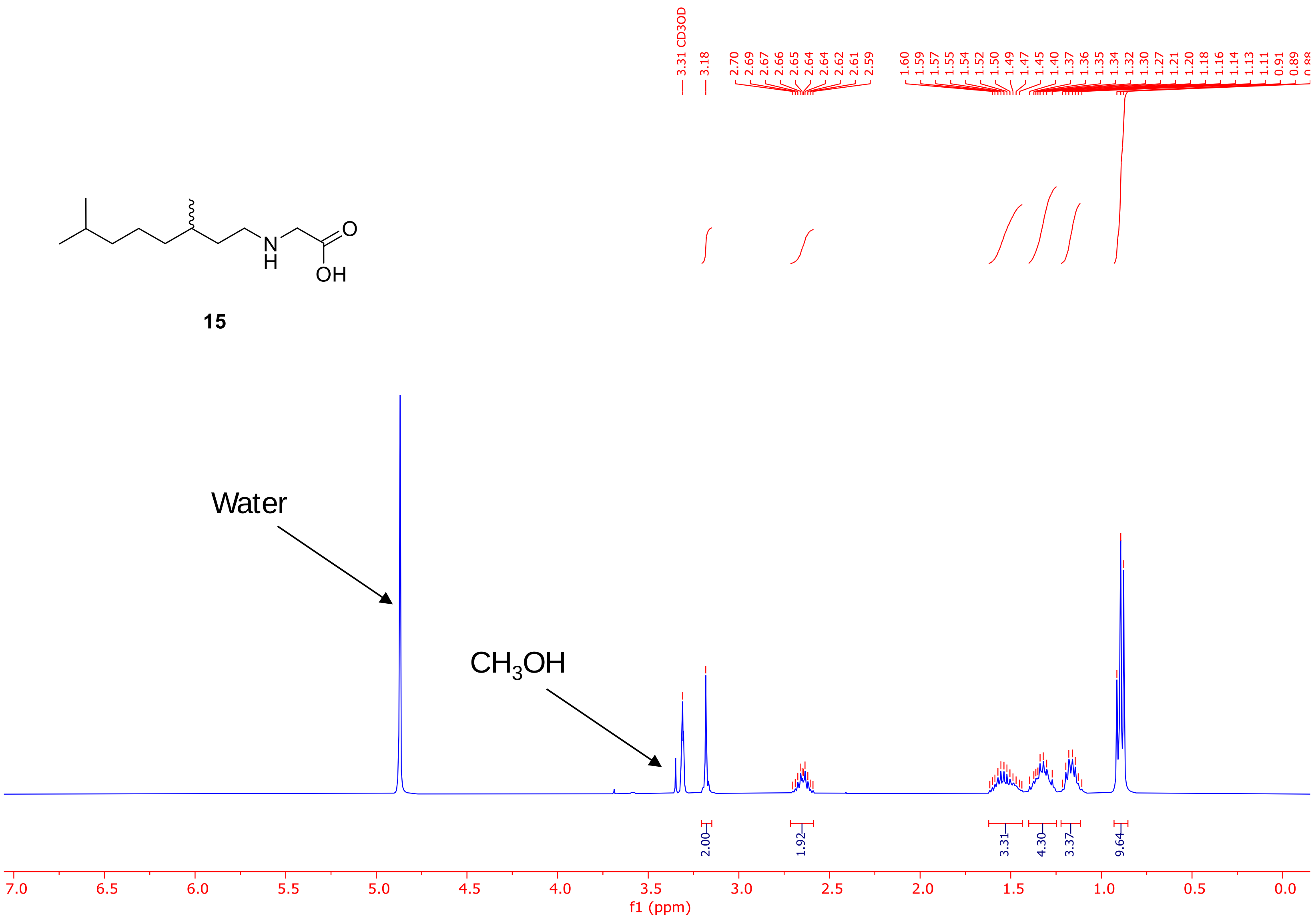

Figure S8: 400 MHz ^{1}H NMR spectrum [recorded in (CD_3OD)] of compound **15**.

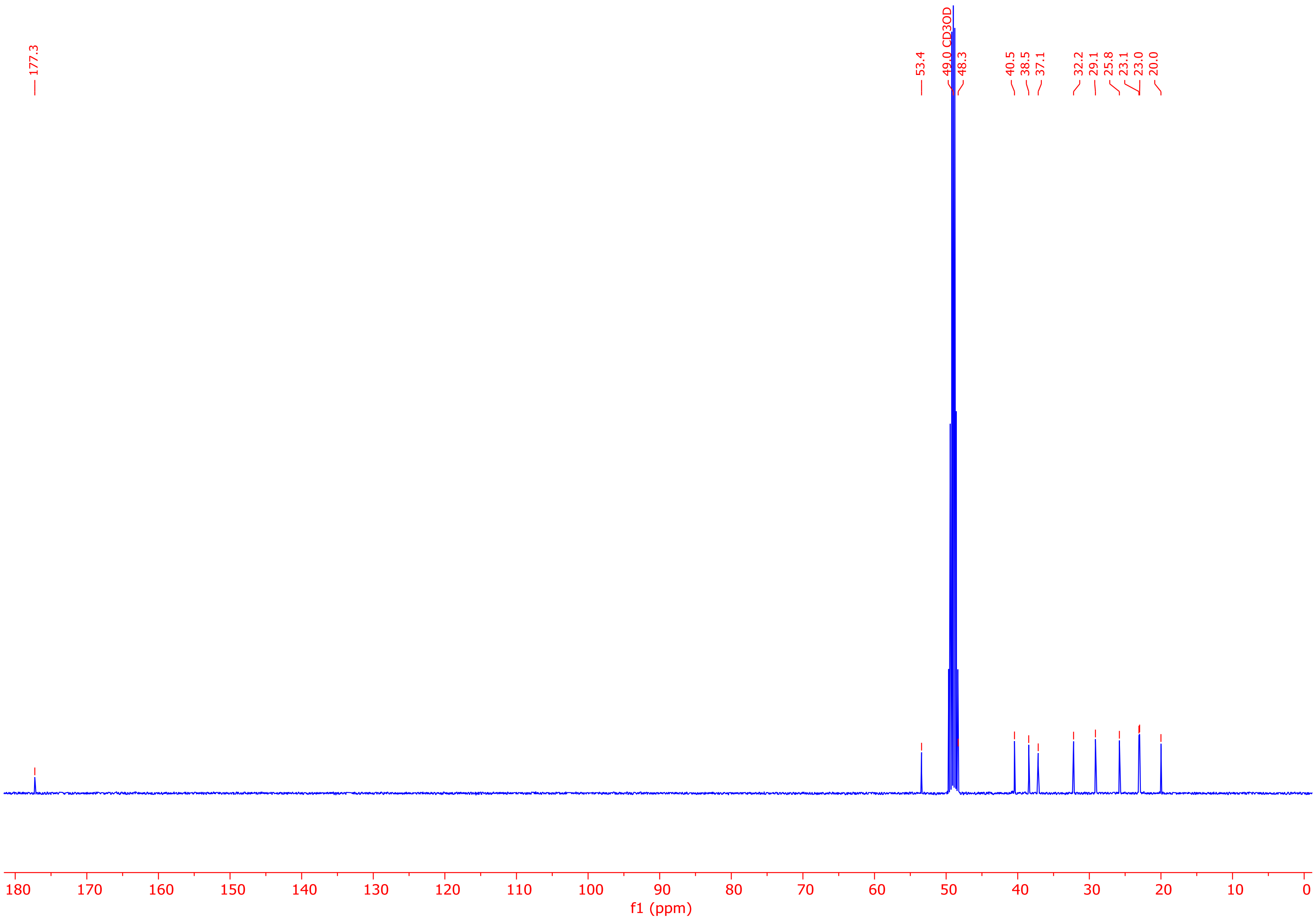

Figure S9: 101 MHz $^{13}C\{^{1}H\}$ NMR spectrum [recorded in (CD_3OD)] of compound **15**.

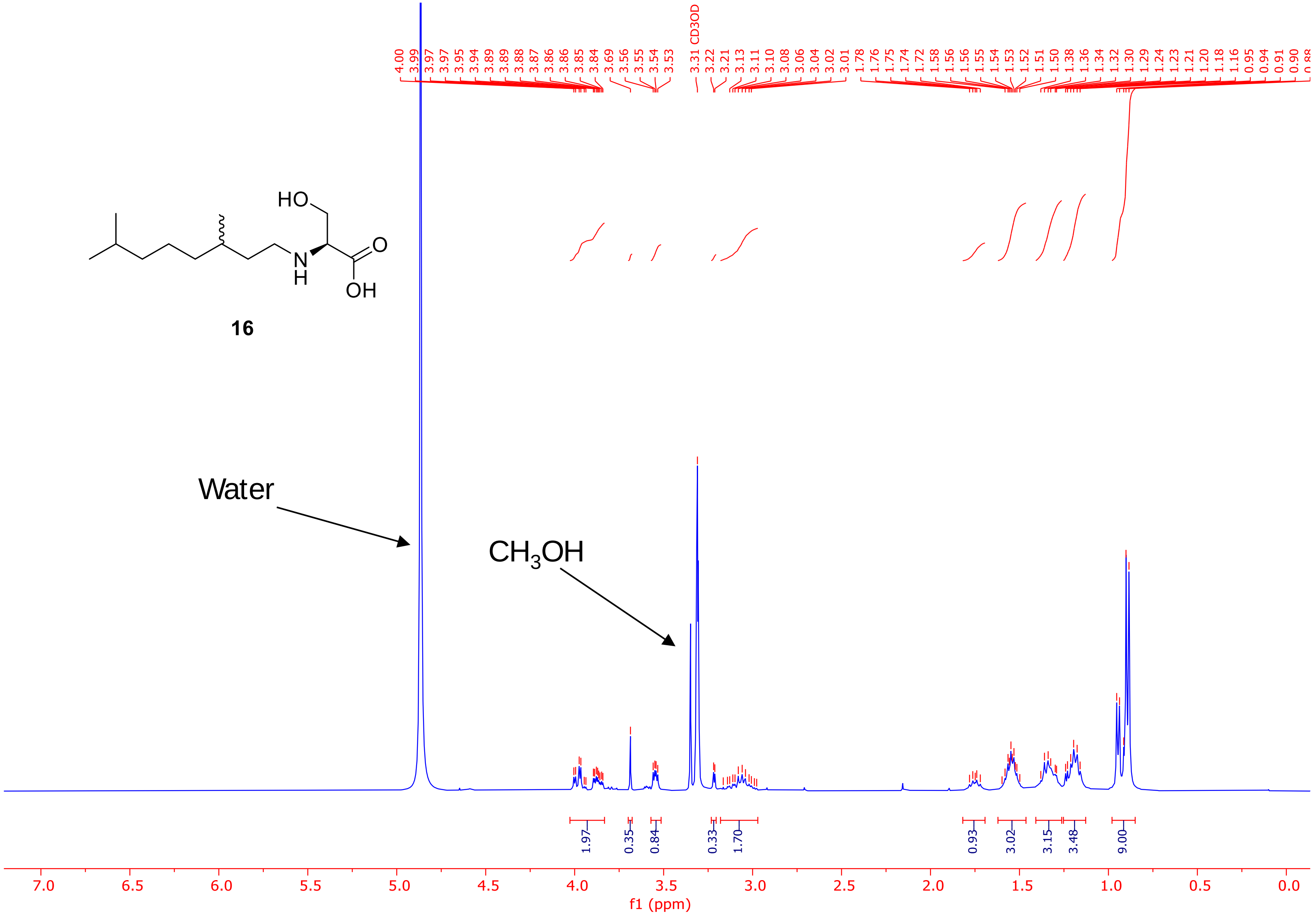

Figure S10: 400 MHz ^{1}H NMR spectrum [recorded in (CD_3OD)] of compound **16**.

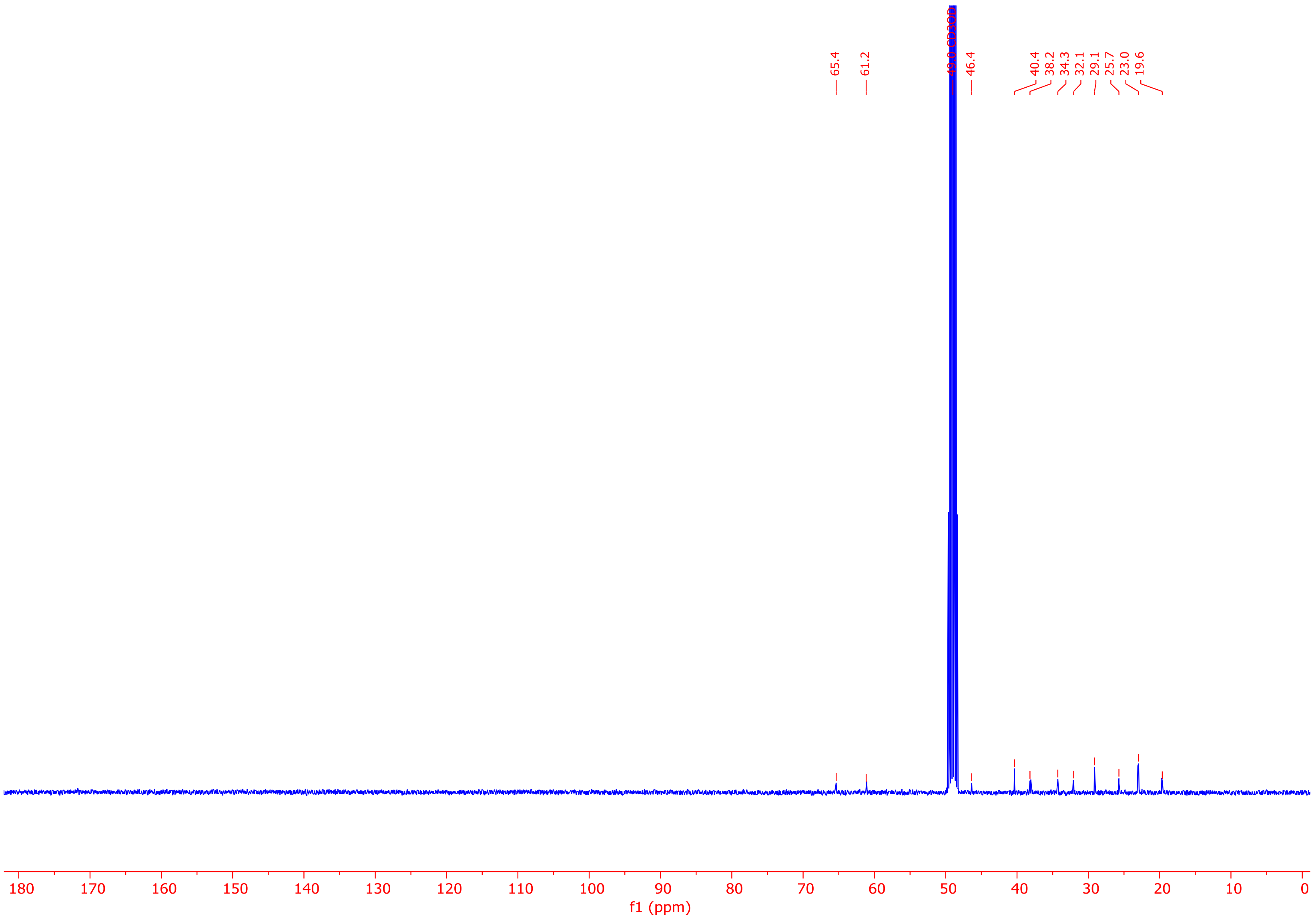

Figure S11: 101 MHz $^{13}C\{^{1}H\}$ NMR spectrum [recorded in (CD_3OD)] of compound **16**.

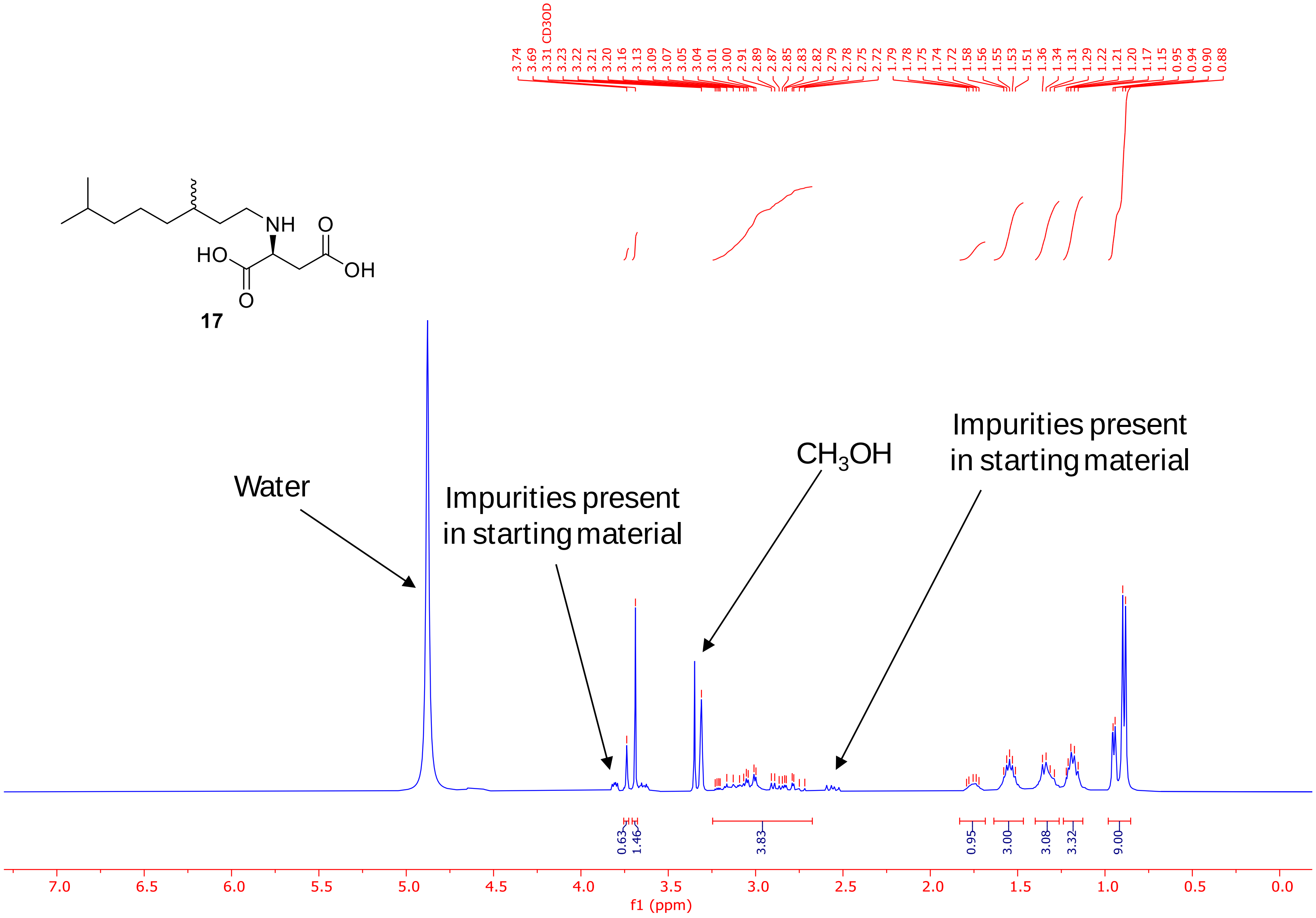

Figure S12: 400 MHz ^{1}H NMR spectrum [recorded in (CD_3OD)] of compound **17**.

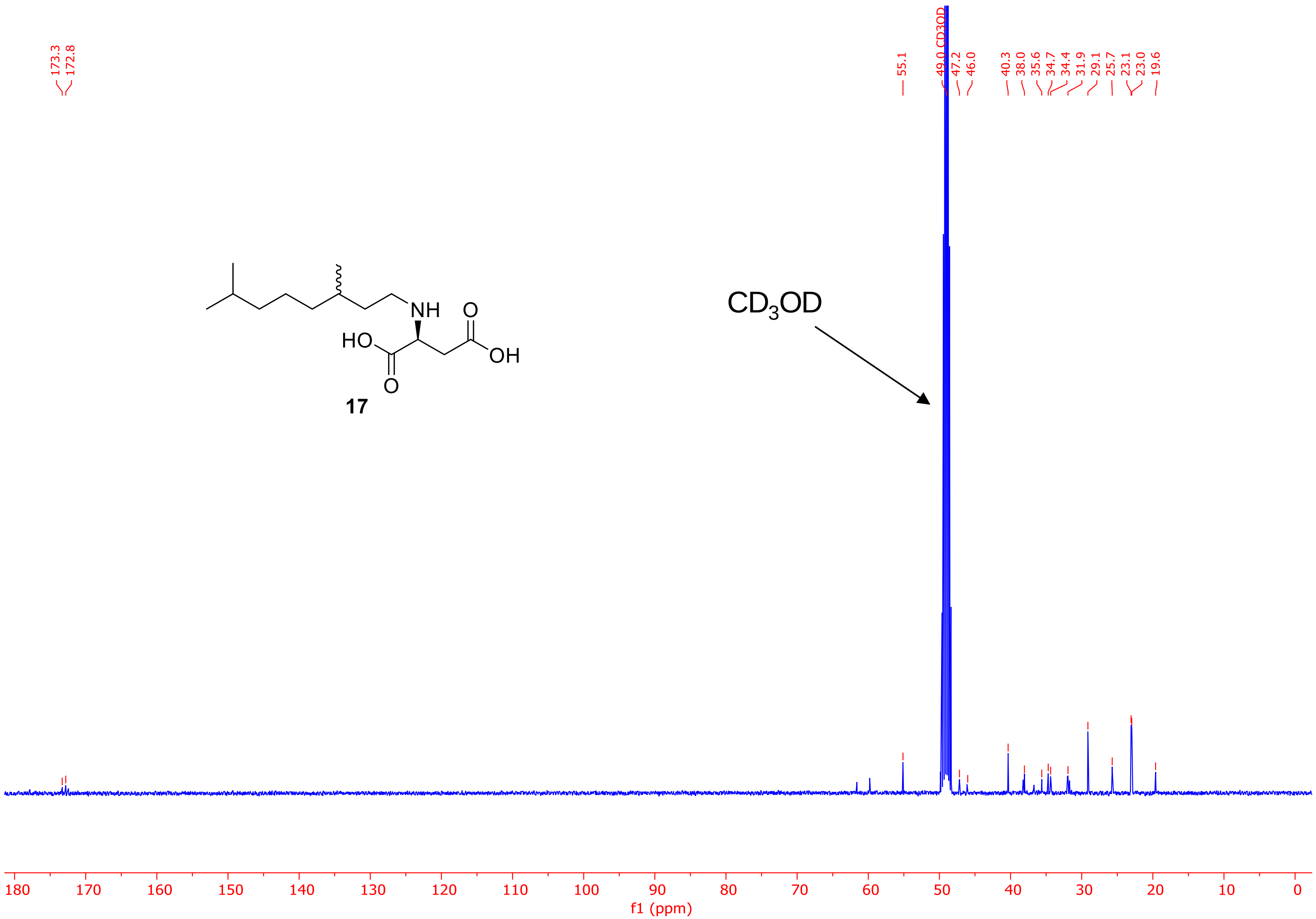

Figure S13: 101 MHz $^{13}C\{^1H\}$ NMR spectrum [recorded in (CD_3OD)] of compound **17**. Additional peaks arising due to isomerism and impurities carried through from the starting material (*L*-aspartic acid).

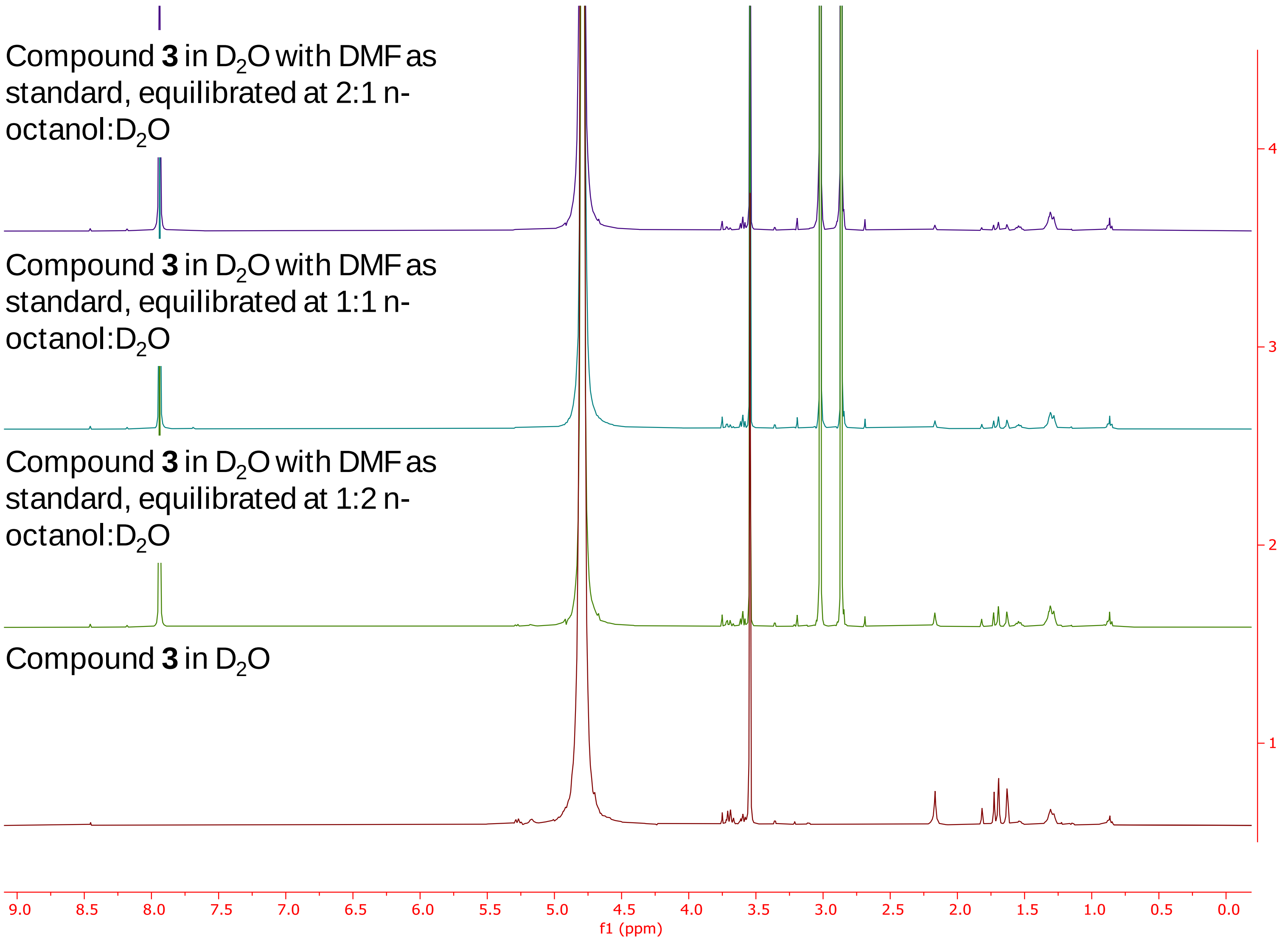

Figure S14: Partial stacked 400 MHz ^{1}H NMR spectra [recorded in (D_2O)] of compound **3** following n-octanol/water partitioning at various solvent volume ratios (as given in the figure) using *N,N*-dimethylformamide as an internal standard.

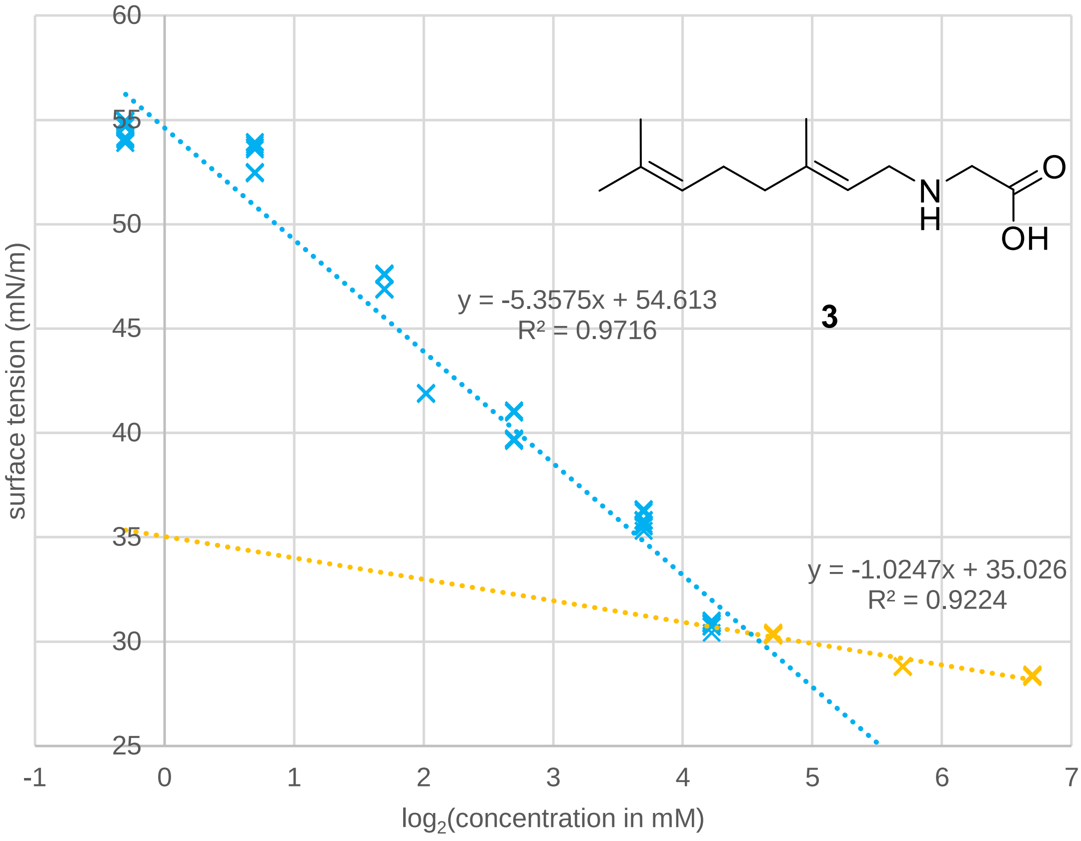

Figure S15: Surface tension measurements for compound **3** recorded at various concentrations.

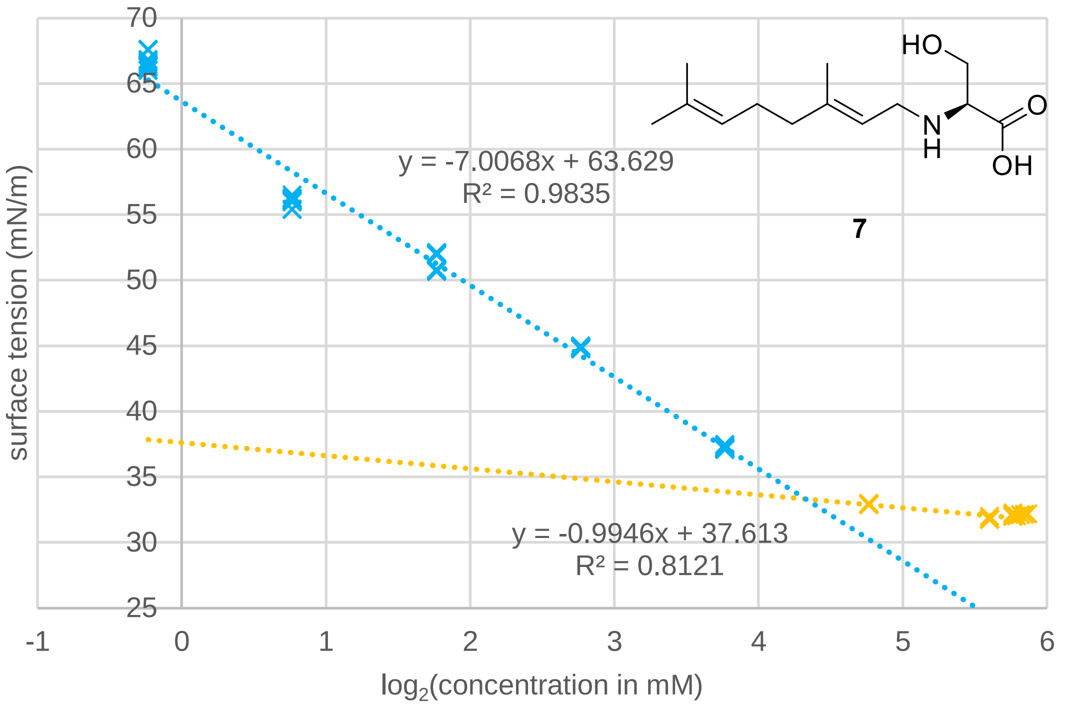

Figure S16: Surface tension measurements for compound **7** recorded at various concentrations.

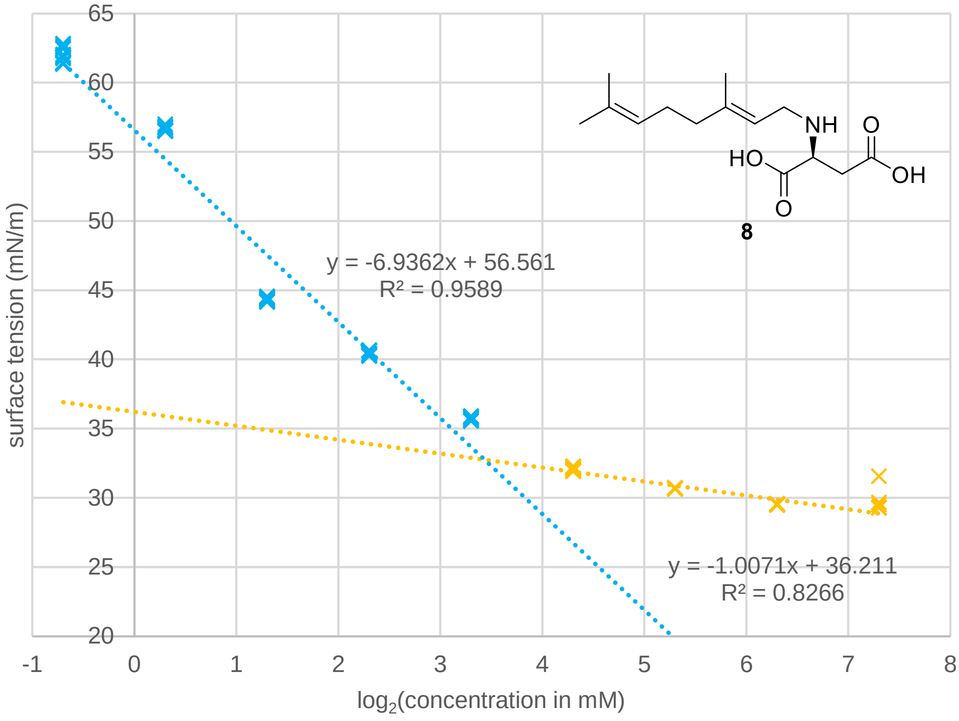

Figure S17: Surface tension measurements for compound **8** recorded at various concentrations.

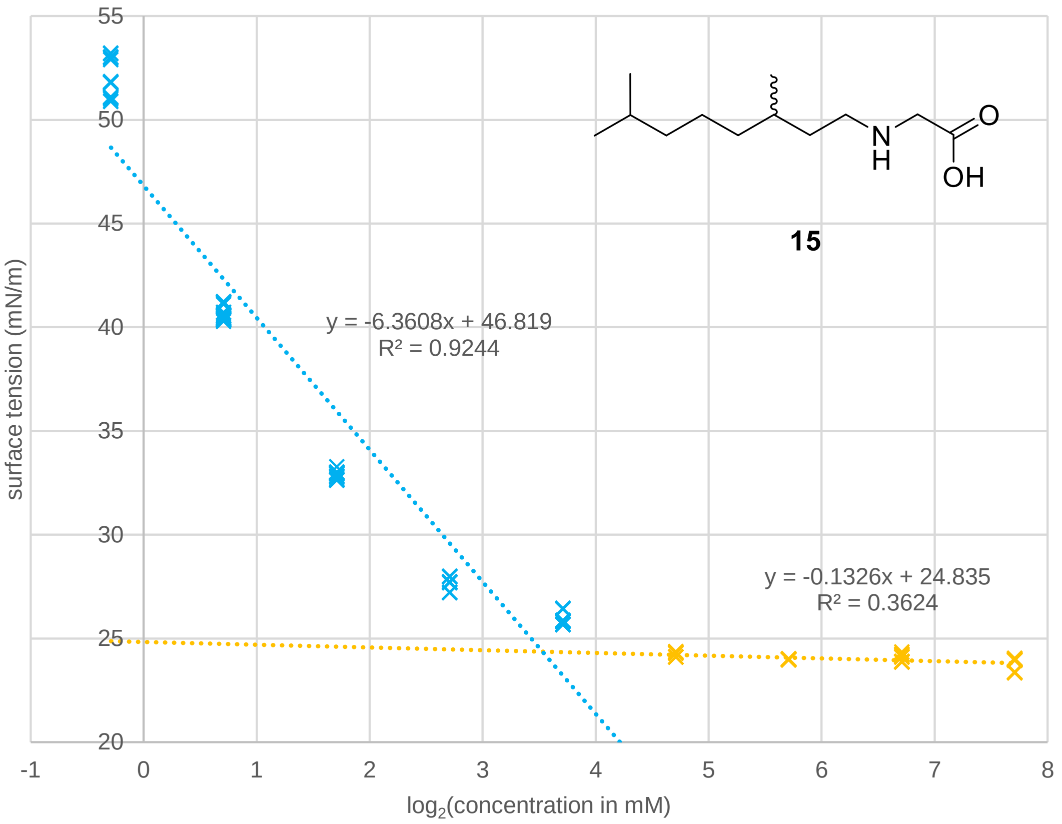

Figure S18: Surface tension measurements for compound **15** recorded at various concentrations.

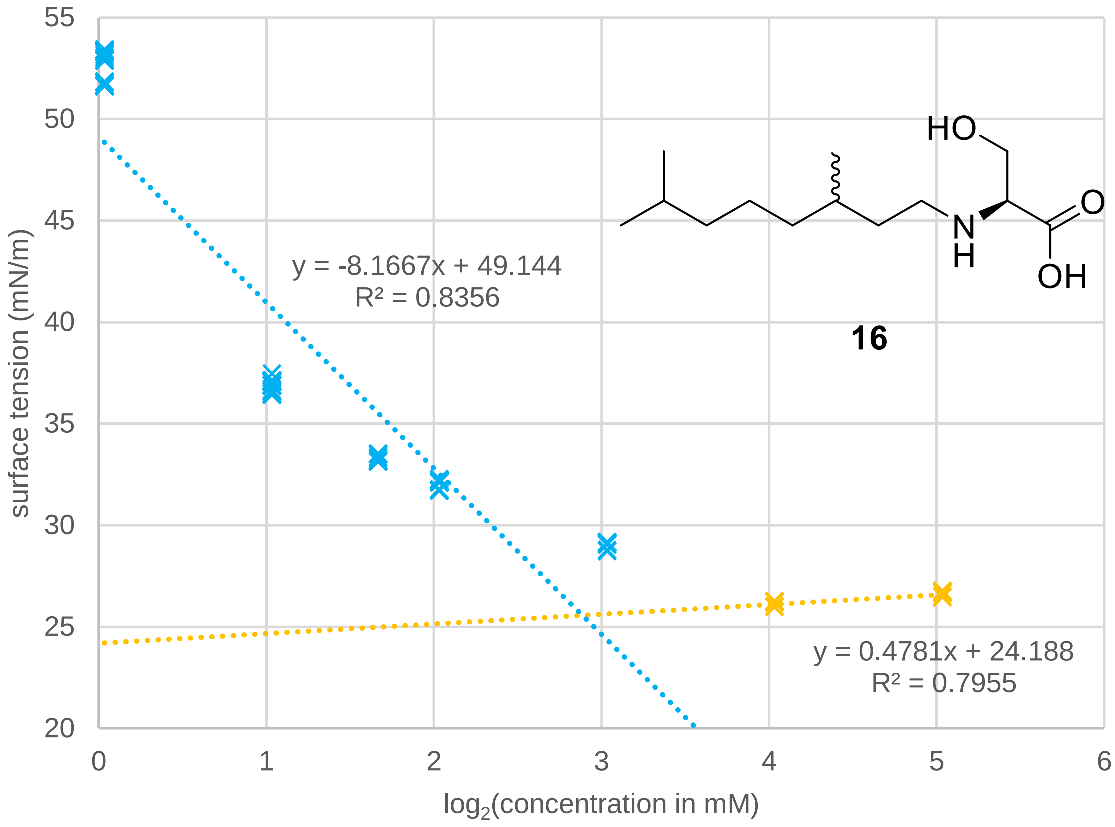

Figure S19: Surface tension measurements for compound **16** recorded at various concentrations.

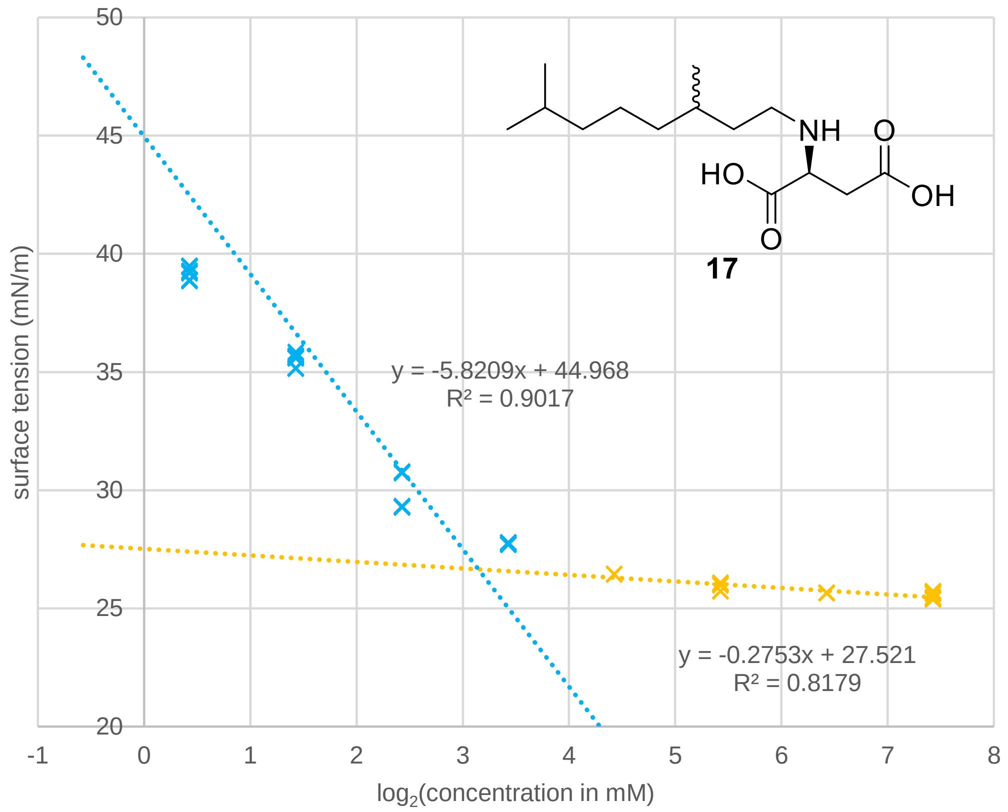

Figure S20: Surface tension measurements for compound **17** recorded at various concentrations.
